# Unlocking the potential of community pharmacies to address hypertension in South Asia: the COPE-BP programme

**DOI:** 10.7189/jogh.16.03005

**Published:** 2026-02-20

**Authors:** Saima Afaq, Helen Elsey, Mohammad Ismail, Khaleda Islam, Rumana Huque, Simon Walker, Catherine Hewitt, Kamran Siddiqi, Aziz Sheikh, COPE-BP Consortium, Cath Jackson, Joao Porto de Albuquerque, Shaista Rasool, Abdul Jalil Khan, Zia ul Haq, Naomi Gibbs, Mehreen Faisal, Faiza Aslam, Waqas Ahmad, Enayet Hussain, Saira Bokhari

**Affiliations:** 1 Department of Health Sciences, University of York, York, UK; 2 Institute of Public Health and Social Sciences, Khyber Medical University, Peshawar, Pakistan; 3 Hull York Medical School, University of York, York, UK; 4 Department of Pharmacy, University of Peshawar, Peshawar, Pakistan; 5 ARK Foundation, Dhaka, Bangladesh; 6 Centre for Health Economics, University of York, York, UK; 7 Nuffield Department of Primary Care Health Sciences, University of Oxford, Oxford, UK

## Abstract

Hypertension is a leading contributor to cardiovascular disease in South Asia, affecting over 40% of adults, of whom most remain undiagnosed or poorly managed. Despite urgent healthcare needs, the already overstretched public primary care systems in low- and middle-income countries, particularly in rapidly growing urban areas, are falling short. Community pharmacies, often the first point of contact for low-income urban residents, represent an opportunity for delivering frontline chronic disease care. The Community-Pharmacies managing hypertension: intervention development and Evaluation in Bangladesh and Pakistan (COPE-BP) programme aims to investigate whether these widely accessed yet under-evaluated community pharmacists are effective and cost-effective, and assess whether the COPE-BP intervention can be successfully integrated into hypertension care pathways in Bangladesh and Pakistan. Beyond evaluating clinical and economic outcomes, COPE-BP seeks to reframe the role of semi-formal providers, including community pharmacies, which are key actors in primary care delivery. Through a stepwise programme encompassing intervention development, a multicentre clinical trial, implementation research, and policy engagement, COPE-BP aims to provide a scalable model for integrating non-traditional providers into national health strategies. In doing so, COPE-BP will help strengthen inclusive, people-centred health systems in LMICs.

Cardiovascular disease (CVD) remains the leading cause of mortality, accounting for over 17.9 million deaths each year worldwide [1]. In South Asia, this burden is particularly stark. Across Bangladesh and Pakistan, CVD is responsible for approximately 1.2 million [2–5] annual deaths. Hypertension is the most important modifiable risk factor for CVD, which affects over 40% [6,7] of adults in Bangladesh, and in Pakistan, around 60–70% [8,9] of individuals with hypertension remain undiagnosed or untreated, and over 60% [10] of those under treatment have uncontrolled blood pressure.

Primary healthcare in urban areas in many low- and middle-income countries (LMICs) is struggling to meet the increasing health needs due to the continued urbanisation and an increasing burden of non-communicable diseases (NCDs) [11–13]. In LMIC cities, public primary care is often absent or, where available, under-resourced primary care facilities have limited opening hours. Combined with perceptions of long wait times and rushed consultations, this limits utilisation by low-income urban residents (LIURs) [14]. Consequently, many patients who could be managed at a primary care level end up in already overcrowded hospitals [11,14]. Private and informal providers have moved in to fill these gaps, and for many, private pharmacies are the first and most frequent point of contact [15,16]. This has led to calls to rethink urban health systems, taking into consideration the motivations and roles of various providers [11–13]. This underscores the urgent need for integrated, community-based solutions to address hypertension and reduce CVD risk. In many LMICs, when formal health systems are under-resourced or inaccessible, individuals increasingly rely on informal or semi-formal providers, including community pharmacies, which become the first points-of-care, especially when free medicines are not available. The Community-Pharmacies Managing Hypertension: Intervention Development and Evaluation in Bangladesh and Pakistan (COPE-BP) programme aims to respond to this need by evaluating if and how the role of community pharmacies, widely accessed yet under-evaluated for chronic disease care, can serve as effective, safe, and scalable platforms for delivering frontline hypertension services to LIURs.

## COPE-BP: STRATEGIC VISION AND WORK PROGRAMME

Guided by the UK Medical Research Council’s framework [17] for complex interventions, COPE-BP takes a stepwise approach that includes intervention development, pilot testing and refinement, measurement of effectiveness, understanding implementation, and assessment of cost-effectiveness for scale-up (**Table 1**). By combining evidence generation with capacity building, COPE-BP aims to offer practical insights into how community pharmacies can strengthen hypertension care in urban South Asia.

**Table 1:** COPE-BP work plan and strategic objectives

Focus	Objective
Intervention development	Co-develop a pharmacist-led, context-specific, hypertension care intervention.
Feasibility assessment	Evaluate feasibility, acceptability, and refine trial protocols via pilot testing.
Effectiveness evaluation	Assess impact on systolic BP through a pragmatic cluster-randomised trial.
Process evaluation	Explore delivery fidelity, mechanisms of impact, and contextual influences.
Economic evaluation	Conduct cost-effectiveness analysis, headroom analysis, value of information analysis, budget impact modelling, and a business case.

BP – blood pressure

### Intervention development

In this phase, we aim to design a contextually appropriate, pharmacist-led intervention for hypertension management that is feasible, acceptable, and aligned with existing health system and regulatory frameworks in Bangladesh and Pakistan. The formative phase begins with geo-spatial mapping and cross-sectional surveys across four urban sites (Bangladesh: Dhaka and Narsingdi; Pakistan: Abbottabad and Peshawar) to assess pharmacy locations, service provision, staff qualifications, and hypertension care practices, highlighting service utilisation and access gaps for LIURs.

We will then explore the real-world functioning of community pharmacies by capturing staff, patients, physicians, and regulators’ perspectives through qualitative research. We will explore issues such as pharmacy registration, ambiguity on their legal scope (*e.g.* blood pressure (BP) checks), and public-private sector tensions, as well as motivations beyond profit, such as professionalism and community service. Based on these findings, we will hold co-design workshops with stakeholders to develop a multi-component intervention. Outputs will include a training curriculum, culturally appropriate patient materials aligned with approved guidelines [18], and a logic model to inform feasibility, pilot and definitive trials, and scale-up.

### Feasibility assessment

In this phase, we assess the feasibility, acceptability, and fidelity of delivering the COPE-BP intervention through community pharmacies and refine trial procedures before a full evaluation. We will conduct a cluster-randomised feasibility trial across pharmacy clusters in both countries, enrolling adults with hypertension. The number of clusters and sample size will be determined based on feasibility parameters and effect sizes observed in the COBRA-BPS trial [19] on hypertension management in Pakistan and Bangladesh, and the feasibility and expected patient volumes identified through initial pharmacy mapping. Randomisation will aim to reduce contamination by incorporating buffer zones, and data will be collected at multiple time points.

In this work phase, we will assess recruitment, retention, fidelity, and data system performance. Our findings will guide refinements to the intervention, training, and supervision. If no major changes are needed, data may be integrated with the full trial. Additionally, we will explore implementation dynamics, including staffing realities, informal practices, and provider motivations, using observations, surveys, and interviews. The phase will also offer early insights into regulatory gaps and delivery constraints in semi-formal provider settings.

### Effectiveness evaluation

A definitive pragmatic trial will include 32 pharmacy clusters and over 1200 participants to measure the impact of the COPE-BP intervention on systolic BP at 24 months. We will inform the projected sample size and cluster estimates by power calculations based on effect sizes observed in the COBRA-BPS trial [19] on hypertension management in Pakistan and Bangladesh. We will refine the final sample size following the feasibility phase, with detailed statistical methods and justification to be provided in the forthcoming full trial protocol. Secondary outcomes will include adherence, patient satisfaction, and quality of life. The control group will receive standard pharmacy services and a hypertension information leaflet, while the intervention group will receive the COPE-BP intervention that includes regular BP checks, lifestyle counselling, medication adherence support, and physician referrals where needed, delivered by trained pharmacists. In the internal pilot phase, we will confirm trial readiness. We will collect data through digital tools, clinical measures, and standardised patient interviews.

### Process evaluation

Within the effectiveness trial, we will conduct a process evaluation to understand how the intervention is delivered and experienced in real-world pharmacy settings. Beyond fidelity, the process evaluation will explore how pharmacists, patients, and supervisors engage with the intervention amidst staffing constraints, digital literacy challenges, gender dynamics, and trust in private providers. Using longitudinal interviews, observations, and fidelity assessments, we will identify what enables or hinders delivery across diverse urban contexts. These findings will guide necessary adaptations for scale-up and inform real-time learning for policymakers, ensuring the model remains responsive to local needs and implementation realities.

### Economic evaluation

Despite increasing interest in leveraging private providers for universal health coverage (UHC), there is limited economic evidence on the value of engaging community pharmacies in delivering chronic disease care in LMIC cities. COPE-BP seeks to address this gap by evaluating whether pharmacist-led hypertension management is not only effective, but also cost-effective and scalable, particularly for LIURs who are often excluded from formal health services.

Using within-trial and modelling approaches, we will estimate the cost per disability-adjusted life years (DALYs) averted and quality-adjusted life year. We will conduct analyses from health system and societal perspectives. Headroom and value of information assessments will guide intervention and research design. We will conduct a business case analysis to assess whether financial incentives sufficiently offset pharmacy delivery costs. Budget impact modelling will inform scale-up potential, supporting integration into UHC frameworks.

### Building capacity for sustainable non-communicable disease care

In Bangladesh and Pakistan, the capacity to conduct complex implementation research and deliver decentralised NCD services remains limited. Most pharmacists and allied health professionals receive little training in patient-centred hypertension care, including accurate BP measurement, counselling, or referral practices. Similarly, community engagement in research and service design is often ad hoc, with few mechanisms for meaningful, sustained involvement of patients and caregivers.

To address these gaps, COPE-BP will involve a multi-level capacity strengthening strategy (**Table 2**). Together, these activities aim to establish a self-sustaining ecosystem for applied NCD research and service innovation, enabling long-term scale-up contingent on proven effectiveness and cost-effectiveness and South–South collaborations.

**Table 2:** Multilevel capacity strengthening strategy for COPE-BP

Capacity area	Activities
**Research capacity**	Five doctoral fellowships (three at the University of York, two at KMU). Training for 30+ early/mid-career researchers in trial design, including process evaluation and health economics, good clinical practice, and implementation science. Mentorship scheme to promote research leadership, especially among LMIC-based and female researchers Supportive tools, including a publication tracker, writing workshops, and a buddy system.
**Community capacity**	- Establishment of the CAPs in each country. Training for CAPs in ethics, advocacy, and research engagement. CAP's involvement in co-design, implementation, and dissemination to ensure cultural alignment and build trust with low-income urban residents.
**Clinical capacity**	Short courses for pharmacists and allied health professionals in BP monitoring, counselling, referral, and gender-sensitive care. Launch of a certificate course in complex RCTs through the KMU Clinical Trials Unit. Data management led by the Bangladesh team with technical oversight from York. Economic evaluations conducted by ARK Foundation in collaboration with the University of York’s Centre for Health Economics.

BP – blood pressure, CAPs – community advisory panels, KMU – Khyber Medical University, LMICs – low- and middle-income countries, RCTs – randomised control trials

### Stakeholder engagement and policy uptake

Community pharmacies remain an under-recognised component of primary care in Bangladesh and Pakistan [20], despite being a first point of contact for many LIURs [21,22]. In both countries, current policies do not formally permit pharmacies to screen or manage hypertension, and regulatory oversight remains fragmented. Pharmacies often operate outside health management information systems, limiting data on their contributions and rendering the needs of LIURs largely invisible in formal planning and governance [23]. There is also a persistent lack of coordination between drug regulators, local governments, and primary health systems, a gap often fuelled by mistrust and differing motivations across sectors.

During the design phase, COPE-BP embedded inclusive stakeholder engagement through a series of individual consultations and multi-stakeholder workshops. Policymakers, pharmacists, physicians, and people living with hypertension helped define the target population, shape recruitment strategies, and prioritise delivery via registered pharmacies with qualified staff. These interactions also identified key training gaps that informed intervention design.

To ensure community ownership, community advisory panels will play a central role in co-design, implementation, and dissemination. To support long-term adoption, COPE-BP is aligning its tools with national guidelines and engaging Ministries of Health, pharmacy councils, and NCD leaders. Research Policy Forums will promote cross-sector dialogue and evidence uptake into NCD and UHC strategies.

Beyond informing current policy, COPE-BP also prompts a wider reflection: should the role of pharmacies be redefined in LMIC health systems? COPE-BP contributes to a growing body of global efforts to integrate community pharmacies into NCD management. By generating context-specific evidence on the feasibility, safety, and impact of pharmacist-led hypertension care, the programme may help further position pharmacies, not merely as retail outlets, but as frontline partners in NCD care, similar to the evolving roles of community health workers in LMICs.

### Anticipated impact and future directions

COPE-BP is well-positioned to generate scalable evidence for reducing the burden of hypertension and CVD in South Asia. By embedding screening, lifestyle counselling, treatment monitoring, and referral within community pharmacies, the programme offers a pragmatic solution to expand primary care access for LIURs.

If proven effective, COPE-BP could reach millions of people with uncontrolled hypertension in Bangladesh and Pakistan – an estimated 35 million adults overall. Even modest improvements in BP control could prevent up to 62 000 deaths annually [24]. These outcomes would contribute directly to national NCD strategies and global targets set by the World Health Organisation Global Action Plan for NCDs [25].

Beyond clinical effectiveness, the programme will offer robust insights into cost-effectiveness, implementation feasibility, and health system integration. The inclusion of a business case analysis will be critical for engaging private pharmacies and informing reimbursement strategies. The evidence generated will be synthesised into policy briefs and shared through targeted stakeholder dialogues to support integration into essential service packages and national pharmacy practice guidelines.

COPE-BP also offers broader learning for LMICs seeking to engage semi-formal providers in NCD management. Through its focus on decentralised, people-centred care, the programme has the potential to inform new models for task-sharing, equity-focused service delivery, and sustainable scale-up of hypertension care.

Looking ahead, COPE-BP aims to catalyse a shift in how community-based health services are designed, implemented, and institutionalised, ensuring that even the most underserved populations have access to effective, continuous hypertension care.
